# *Echinacea purpurea* (L.) Moench: Biological and Pharmacological Properties. A Review

**DOI:** 10.3390/plants11091244

**Published:** 2022-05-05

**Authors:** Cristina Burlou-Nagy, Florin Bănică, Tünde Jurca, Laura Grațiela Vicaș, Eleonora Marian, Mariana Eugenia Muresan, Ildikó Bácskay, Rita Kiss, Pálma Fehér, Annamaria Pallag

**Affiliations:** 1 Doctoral School of Pharmaceutical Sciences, University of Oradea, 410087 Oradea, Romania; cristina_burlou@yahoo.com; 2 Department of Pharmacy, Faculty of Medicine and Pharmacy, University of Oradea, 410028 Oradea, Romania; florinbanica1@gmail.com (F.B.); jurcatunde@yahoo.com (T.J.); laura.vicas@gmail.com (L.G.V.); marian_eleonora@yahoo.com (E.M.); 3 Department of Preclinical Disciplines, Faculty of Medicine and Pharmacy, University of Oradea, 410068 Oradea, Romania; marianamur2002@yahoo.com; 4 Department of Pharmaceutical Technology, Faculty of Pharmacy, University of Debrecen, H-4032 Debrecen, Hungary; bacskay.ildiko@pharm.unideb.hu (I.B.); feher.palma@pharm.unideb.hu (P.F.); 5 Department of Pharmacology and Pharmacotherapy, Faculty of Medicine, University of Debrecen, H-4032 Debrecen, Hungary; kiss.rita@med.unideb.hu

**Keywords:** *Echinacea purpurea* (L.) Moench, bioactive compounds, immunomodulatory, cannabinomimetic, anti-inflammatory, antiviral, antimicrobial, antioxidant effect

## Abstract

*Echinacea purpurea* (L.) Moench (EP)is a perennial herbaceous flowering plant, commonly known as purple coneflower and it belongs to the Asteraceae family. The Echinacea genus is originally from North America, in the United States, and its species are widely distributed throughout. There are nine different species of Echinacea, but only three of them are used as medicinal plants with wide therapeutic uses: *Echinacea purpurea* (L.) Moench, *Echinacea pallida* (Nutt.) Nutt. and *Echinacea angustifolia* DC. Several significant groups of bioactive compounds with pharmacological activities have been isolated from Echinacea species. Numerous beneficial effects have been demonstrated about these compounds. The immunomodulatory effect was initially demonstrated, but over time other effects have also been highlighted. The present review gives a comprehensive summary of the chemical constituents, bioactive compounds, biological effects and therapeutical uses of purple coneflower. Research shows that such a well-known and recognized species needs to be further studied to obtain efficient products with a guarantee of the safety.

## 1. Introduction

Nature has always been a great source of therapeutic substances, providing us with various medicinal plants that produce valuable phytochemicals. 

Medicinal plant use dates back to remote times and it is therefore believed to be the genesis of modern medicine. In addition, chemicals generated from plants are and will continue to be a valuable source of molecules for pharmaceuticals [1]. In the past, folk observations and experience were the guiding principles in the use of herbs, but today their active ingredients, their action mechanisms and their usage based on principles of evidence-based medicine have been discovered.

Phytotherapy is widely utilized in the treatment and prevention of a variety of medical conditions and is well-known among the general populace [2].

Plant-based pharmaceuticals now represent about 30% of the drugs market made up of necessary medications, while the remaining 11% is made up of non-essential drugs [3,4]. 

This evaluation's primary goal is the synthesis of the data from the specialized literature regarding the bioactive compounds, chemical composition, pharmacological and biological properties of *Echinacea purpurea* (L.) Moench to highlight the possibilities and perspectives for future studies, and to obtain safe and pharmacologically effective products.

The species of the genus Echinacea occupy an important place among medicinal plants, are native to North America and belong to the Asteraceae family. Echinacea comes in nine distinct species, but just three are utilized as medicinal herbs with wide therapeutic uses: *Echinacea purpurea* (L.) Moench, *Echinacea pallida* (Nutt.) Nutt. and *Echinacea angustifolia* DC [5,6].

A large number of species in the Asteraceae family have been utilized for therapeutic purposes in comparison to other plant families because of the availability of chemicals with a broad spectrum of therapeutic properties, as well as the fact that the Asteraceae family of plants is one of the most prominent and well-known [7,8].

**Figure 1:**
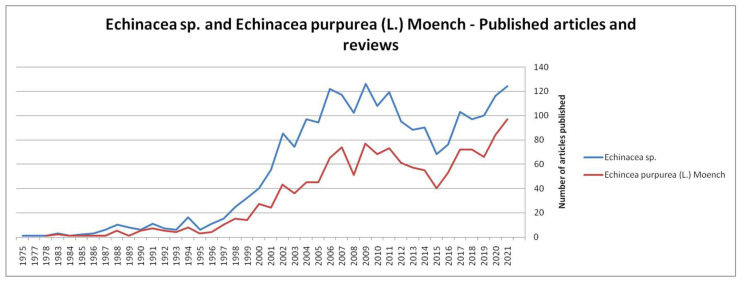
Evolution of the number of articles and reviews about Echinacea sp. and *Echinacea purpurea* (L.) Moench [9,10].

Figure 1 shows the evolution over time of publications on the genus Echinacea and the species *Echinacea purpurea* (L.) Moench. There has been an increase in scientific research on these species over time until 2009, after which there was a slight decrease until 2015, followed by an increase again until today [9,10].

*Echinacea purpurea* (L.) Moench is a perennial plant, 100–150 cm tall, vigorous and herbaceous. The roots and rhizome are highly developed. *Echinacea purpurea* (L.) Moench is a perennial plant, vigorous, 100–150 cm tall and herbaceous. The roots are cylindrical, brownish-gray on the exterior and white on the interior. The aerial stem is branching and has rough hairs and reddish-brown patches, giving it the appearance of a bush. Linear-lanceolate leaves with three arching ribs and rough hairs are entire, 3–6 cm wide. It produces a rosette of leaves during the first year of cultivation and blooms only in the second year [11,12,13].

The flowers are grouped in inflorescences, the terminal anthodes are pink, pale red to deep red. At the base of the inflorescence there are sharp bracts that, when mature, lignify and become spiky. The sterile ray flowers are pink, located at the edge of the inflorescence, and the ligules are 5–7 cm long and 0.5 cm wide. The disk flowers are tubular, orange-brown, bisexual and are placed inside the inflorescence. The fruits are achene, white-gray, edged, with four edges and teeth at the top. It blooms in June–July [14,15,16,17,18]. Invasive weeds are a common problem for Echinacea crops. Chemicals such as herbicides are one of the most efficient weed control tactics. Herbicides are harmful to the Echinacea's physiological and morphological processes [19].

## 2. Methodology

**Figure 2:**
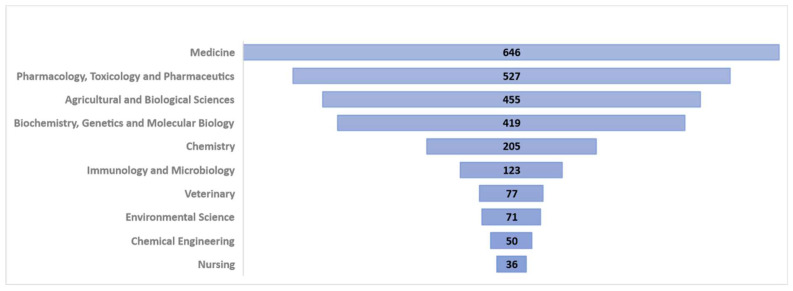
Publications by scientific categories indexed in Scopus [9].

This review was based on experimental research from a literature survey report on Echinacea species. The review involved a literature search and a selection was includedof published scientific evidence on the subject of *Echinacea purpurea* (L.) Moench. There were no constraints on the publication date of the selected articles, which included both recent and older works, to gather all essential details for the research. Furthermore, the most relevant databases for the aim mentioned above were the Web of Science Core Collection and Scopus. Figure 2 shows the distribution of the articles included in the Scopus database by scientific categories [9].

**Figure 3:**
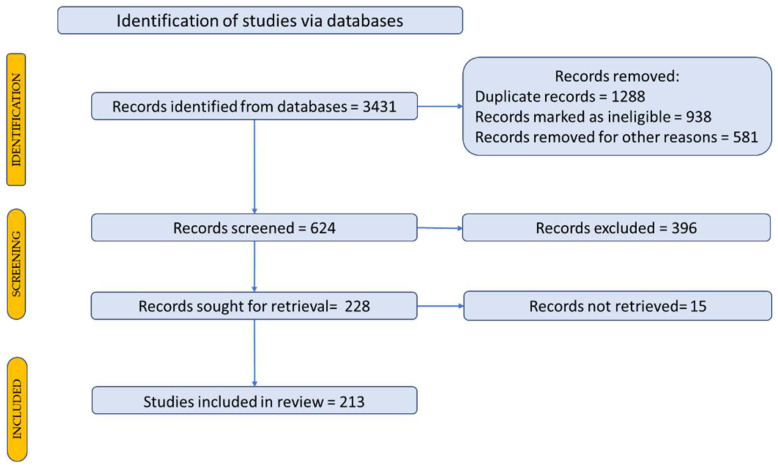
Prisma flow diagram for a description of the selection process of the bibliographic sources.

To identify scientific articles published up to December 2021, the articles were searched using representative keywords to locate the primary data, outcomes and papers in the field. To search for articles, the following keywords were used: *Echinacea* and *Echinacea purpurea* (L.) Moench. A total of 3431 scientific articles were identified. A Prisma flow diagram was used for a description of how to select the studies, with the articles included in the review, as shown in Figure 3 [20,21].

From the beginning, we established the eligibility criteria for the selection of studies. 

The inclusion criteria included articles with extensive studies on the chemical composition and bioactive compounds of Echinacea species, especially EP, studies on the therapeutic use of EP, studies on the antioxidant, immunomodulatory, anti-inflammatory, antimicrobial, antiviral and pharmacological effects, and on side effects and toxicity.

The exclusion criteria were as follows: if the subject area is from chemical engineering and veterinary, we found only the published abstract, not the whole study, and studies that were not published in English.

Starting from this number of articles, we carried out the selection process for choosing the most relevant articles for this research. We discovered the most important data/results/papers in the field, resulting in 213 articles.

### 2.1. Bioactive Compounds of Echinacea purpurea (L.) Moench

**Figure 4:**
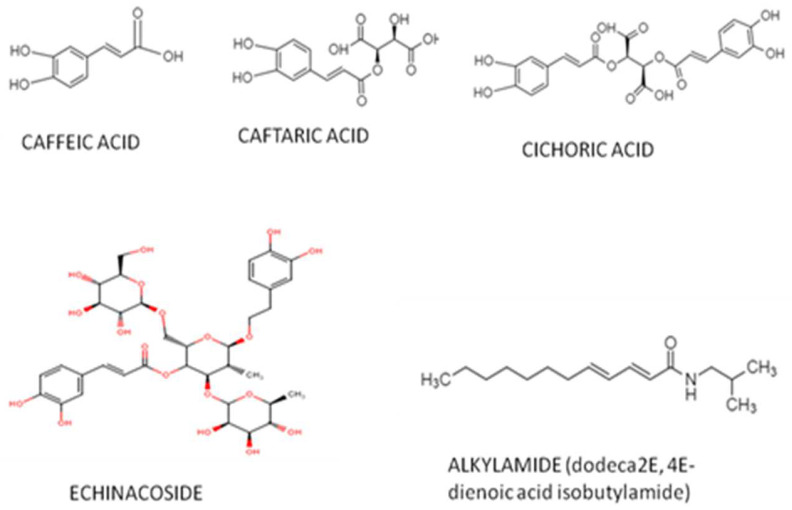
Structure of major compounds.

Several significant groups of bioactive compounds, with pharmacological activities, have been isolated from Echinacea species. The most important components of *Echinacea purpurea* (L.) Moench are alkylamides, polysaccharides, glycoproteins, flavonoids and phenolic compounds, which include [22] derivates of caffeic acid, like caffeic acid, chicoric acid, caftaric acid, chlorogenic acid and echinacoside, Figure 4, [23,24], whose amounts vary based on the plant's sections. In addition to these components, we also identified thatphylloxanthobilins, β-phellandrene, acetaldehyde, dimethyl sulfide, camphene, hexanal, α-pinene and limonene are present in all plant tissues, regardless of species. Fatty acids, aldehydes and terpenoidsare constituents whose presence depend on the parts of plants used [25,26,27,28,29,30,31,32,33,34,35,36,37,38].

The chemical components responsible for the immunomodulatory activities of purple coneflower roots are glycoproteins, alkylamides and polysaccharides [39]. Glycoproteins are proteins and carbohydrate chains that have a role in a variety of physiological activities, including immunology. Alkylamides are a kind of chemical found in genus Echinacea (Asteraceae) that has been demonstrated to have high bioavailability and alsoimmunomodulatory properties. Structurally, they have a common feature of an amine bond and usually contained an aliphatic chain of polyunsaturated fatty acids connected to a short-chain amine. Polysaccharides are complex carbohydrate polymers made up of more than two monosaccharides. The Asteraceae family contains important polysaccharides, pectins, arabinogalactans and inulin. Bioactive polysaccharides may substantiate a part of the traditional uses of these species. 

EP supplements are, in general, sold as encapsulated tablets containing aerial parts or dried roots or as tablets containing extruded material from pressed plants or ethanol extracts.

The above-ground parts of the plant contain smaller amounts of volatile oils and pyrolizid alkaloids such as tussilagin and isotussilagin than the root [40].

The most important derivative of caffeic acidof the *Echinacea purpurea* (L.) Moench species is considered to be chicoric acid, the most predominant phenolic component in the root and petiole [41,42,43,44,45]. Chicoric acid is the most abundant phenolic component in the root and petiole of *Echinacea purpurea* (L.) Moench. These antioxidant and antibacterial compounds can help the immunological system of the body to function better. However, caffeic acid derivative concentrations will vary according to EP species, organ type, growing conditions and environmental factors.

Using the HPLC method, some studies demonstrated the retention of caffeic acid derivatives in dried EP. It was reported that chicoric acid accounted for 63% and 67%, respectively, of the relative peak area for aerial sections. By using the HPLC method, caffeic acid cannot be detected in all dried flowers. In contrast, the caffeic acid content was 8–18% of the measured relative peak area [46,47,48]. Chicoric acid (71.45%) is the most predominant caffeic acid derivative in flowers, followed by caffeic acid (23.25%) [49].

Echinacoside was discovered to have a variety of pharmacologically important benefits on human health, particularly neuroprotective and cardiovascular effects [35,50]. Echinacosite is a caffeic acid derivative found in the flower at a concentration of 1.45% [48]. In addition to these substances, Echinacea species contain flavonoids, polyacetylenes and alkaloids [51,52].

The constituents that are also important, isolated from the extracts of *Echinacea purpurea* (L.) Moench leaves, are phylloxanthobilins. The breakdown of chlorophyll produces these natural tetrapyrrole compounds. Phylloxanthobilins were identified in the leaves of deciduous trees about 10years ago and are currently considered a compound class with great bioactivity potential, which has yet to be studied. However, there have been no reports of phylloxanthobines being found in sections of a medicinal plant utilized in pharmaceutical formulations until now [53,54].

β-phellandrene was discovered to be prominent in the roots of EP, but lacking in all tissues of *Echinacea pallida* Nutt, in one research study using gas chromatography/mass spectrometry. α-Myrcene was found in large concentrations in all three Echinacea species' flowers, leaves and stems, but was missing from the roots of *Echinaceae angustifolia* and *Echinacea purpurea* (L.) Moench and was only in small concentrations in the roots of *Echinacea pallida Nutt*. Acetaldehyde, dimethyl sulfide, camphene, hexanal, α-pinene and all plant tissues contain limonene, regardless of species. Dimethyl sulfide has been identified in trace amounts in all species' leaves, stems and flowers; nonetheless, it was the most abundant ingredient in *Echinacea pallida* roots and, second, was the most abundant component in *Echinacea angustifolia* and *Echinacea purpurea* (L.) Moench roots. Butanals and propanals, in particular, are aldehydes, comprising 41–57% of root tissue headspace and 19–29% of leaf tissue headspace, and just 6–14% of the headspace between the bloom and the stem tissue. Terpenoids such as α–myrcene, β– and α–pinene, ocimene, camphene, terpenene and limonene constitute between 82–91% of the headspace of stems and flowers, 46–58% of the leaf tissue's headspace and 6–21% of the roots. Furthermore, there are 14 hydrocarbons, 12 alcohols, 7 esters, 6 ketones and 7 other chemicals found [55,56,57]. In another study, we identified compounds in an n-hexane extract of *Echinacea purpurea* (L.) Moench such as fatty acids comprising 9,12-octadecadienoic acid (linoleic acid), hexadecanoic acid (palmitic acid) and octadecanoic acid (stearic acid), accounting for 25.8% of the extract, and after that come the next long-chain hydrocarbons (14.6%) and sterols (13.9%) [58].

EP has also been prepared for use as a topical treatment for skin and wound inflammation. In addition, Echinacea products are licensed in Europe to heal infections of the upper respiratory tract and wound healing [59,60,61,62].

Many bioactivities of EP have been discovered in modern pharmacological investigations, including immunomodulatory, anti-inflammatory, antioxidant, antiviral and antifungal activities [63,64].

Chronic arthritis, cancer, antimicrobial action, persistent fatigue syndrome, HIV infection, a range of skin ailments, wounds and chronic pelvic infections were all mentioned as potential therapeutic uses for EP [65].

Preparations containing EP are among the best-selling herbal medications in Europe and the United States [60,66]. EP supplementation may decrease the severity and duration of acute respiratory tract infections, according to current research; however, no studies have been identified using Echinacea to prevent or treat the SARS-CoV virus infection [67].

The immunomodulatory effects of Echinacea species are of primary concern for research, especially those related to upper respiratory tract infections. Discoveries made recently have also shown that certain standardized preparations of Echinacea have strong antiviral, antifungal, antimicrobial, anti-inflammatory, antioxidant and psychoactive activities. Given the available data, preparations obtained from Echinacea are well-tolerated by the human organism [68]. Therefore, further investigations are needed to ensure the quality and safety of the various preparations of Echinacea sp. [69]. Echinacea sp. can cause minor side effects; it should be considered if the patient who is receiving Echinacea sp. preparations is allergic to *Ambrosia artemisifolia L.* or other species of the Asteraceae family. *Echinacea* sp., like many other Asteraceae plants, includes phototoxic polyacetylene compounds that can be inactivated with minimal processing [70,71].

It is important to note that during preservation, enzymatic processes have the potential to degrade bioactive substances as a result of long-term storage from collection to marketing, leading in compositional changes. 

Stuart and Wills (2000) studied the alkylamide and chicoric acid content of ground and dried *Echinacea purpurea* (L.) Moench roots for 60 days, Table 1 [71,72].

### 2.2. Biological and Pharmacological Effects of Echinacea purpurea (L.) Moench

Currently, the number of herbs that are subject to scientific studies is increasing. The well-known medicinal plants are intensively studied to obtain the most accurate data about the chemical composition, the pharmacological effects and the safety of use in therapy [73].

Table 2 summarizes the most important components identified in *Echinacea purpurea* (L.) Moench and the scientifically proven biological and pharmacological effects, according to the literature. It can be seen that most of the demonstrated effects are common to several compounds, such as the immunomodulatory, antioxidant or antimicrobial effects.

**Figure 5:**
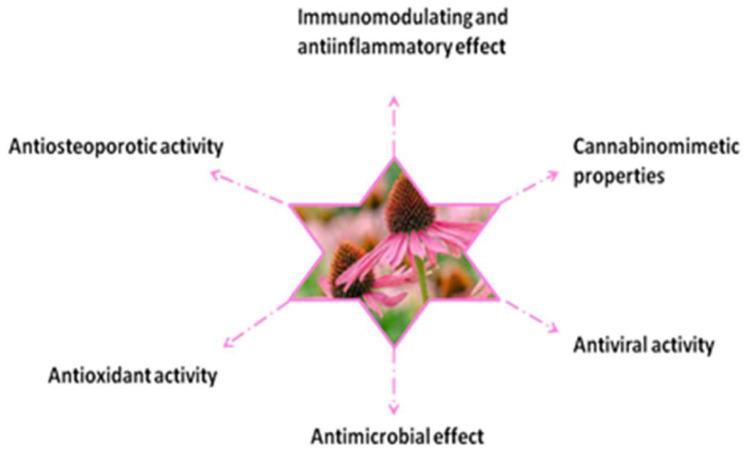
Properties of *Echinacea purpurea (L.) Moench*.

Following the study of the literature, we identified a large number of scientifically proven therapeutic properties for *Echinacea purpurea (L.) Moench* (Figure 5).

#### 2.2.1. Immunomodulating and Anti-Inflammatory Effect

Three pathways are responsible for the immunostimulant action of *Echinacea* species or preparations: phagocytosis activation, fibroblast stimulation and increased respiratory activity all contribute to increased leukocyte motility [127,128]. Several in vivo investigations have been conducted on the immunomodulatory and anti-inflammatory properties of *Echinacea purpurea* (L.) Moench and suggest that administration of the plant boosts innate immunity and bolsters the immune system's ability to fight pathogenic infections by activating neutrophils, macrophages, polymorphonuclear leukocytes (PMN) and natural killer (NK) cells [129].The roots and the aerial part of EP include caffeic acid derivatives, alkamides, ketoalkenes, polysaccharides and glycoproteins; they are thought to be responsible for the immunostimulatory and anti-inflammatory properties [29,130,131,132,133]. In macrophages, phagocytosis and cytokine production (increased tumor necrosis factor -α (TNF-α), interleukin 1 (IL-1) and interferon beta (IFN-β) were established following treatment with Echinacea extract; increased leukocyte mobility and NK cell activation were also demonstrated reasonably in animals and humans [134,135,136].

These advancements will allow for the development of a more extensive range of Echinacea products to keep up with the ever-growing market potential.

Many studies have been done on Echinacea for conditions such as the common cold and other upper respiratory tract infections or its immunostimulatory action, but there is much less research on the use of Echinacea for other purposes, such as preventing and curing dermatological conditions such as atopic eczema and pruritus. About 2–10% of adults and 15–30% of children suffer from atopic eczema, also known as atopic dermatitis, and this is perhaps the most widespread skin condition that causes inflammation [137]. This chronic inflammatory skin condition frequently manifests itself as a rash with extreme itching [138]. In the case of atopic eczema, there is still a demand that has not been satisfied for topical anti-inflammatory and anti-pruritic drugs with an outstanding safety profile [139].

Oláh A. et al. performed in vivo and in vitro studies on human subjects and cell culture, demonstrating that a root extract of *Echinacea purpurea* (L.) Moench formulated in a water/oil (W/O) emulsion exerts relevant anti-inflammatory effects clinically, enhances the epidermal lipid barrier and soothes skin complaints of patients with atopic dermatitis. Thus, this W/O emulsion containing EP extract has been shown to be a very promising product for the daily medical care of the skin in subjects with atopic dermatitis [140].

Many inflammatory skin conditions cause pruritus, including atopic eczema and psoriasis, which is a frequent and predominant symptom [141]. In one study, the antipruritic effects on human subjects were evaluated using two types of emulsions and a shampoo containing a lipophilic extract of root of *Echinacea purpurea* (L.) Moench. Finally, the use of an emulsion decreased dryness, erythema and pruritus considerably [142]. Inflammation is caused by the production of fibrin clots and the degranulation of aggregate platelets, which produce the chemotactic molecules required for the recruitment of leukocytes, especially neutrophils, as well as bone-marrow-derived stem cells or wound fibrocytes. Elastase and collagenase are produced by neutrophils, as well as TNF-α and IL-1, which attract fibroblasts and epithelial cells [143]. Echinacoside is a caffeine conjugate of Echinacea that has been shown to have antihyaluronidase effects. Rousseau et al. are the ones who investigated the effects of Echinacea to heal wounds and the effect of hyaluronuronidase was tested on the vocal folds in the larynx of pigs. The test was performed by random topical applications of different concentrations (1, 200, 600 or 300 mg) of standardized Echinacea on the injured side. A favorable result of antihyaluronidase therapy on the healing of acute vocal wounds has been obtained [144].From the beginning of humanity [145], it was also acknowledged that the antioxidant, anti-inflammatory and antimicrobial activity of herbal compounds aids the healing process [2] because of the active biological compounds, each of them with specific structure and action [146]. Therefore, in Chinese medicine, *Echinacea purpurea* (L.) Moench herba, is used as a treatment for trauma and eczema and improves the skin's barrier function and skin immunity. An in vitro study using a keratinocyte cell culture was performed and phototoxicity tests were performed. The results showed that it has a good moisturizing and water-blocking effect on the skin and does not cause skin irritation, phototoxicity or side effects [147]. According to the 2008 evaluation report, 10–20 g of liquid per 100 g of preparation was utilized in products on the European market. However, this could not be proven in 2013 and there is a tradition of utilizing that range; for example, there is information about ointments containing 10% of the squeezed juice of EP plants in the Rote list of 1961.It is considered that the dry juice equivalent to the juice mentioned above may be equally effective for traditional cutaneous medical products. According to information exchanged between competent authorities in 2013, 16 g of juice expressed per 100 g of preparation is utilized in medications licensed on the European market [148]. Viruses and bacteria cause many skin diseases, and some of them can be cured topically using *Echinacea purpurea* (L.) Moench.

At the same time, it has been shown that a cream containing *EP* extract improves the symptoms of eczema and helps to repair the thin and protective outer layer of the skin. However, Echinacea extract has a shorter shelf life, making it difficult to incorporate into commercial skin care products [147,148].

#### 2.2.2. Cannabinomimetic Properties

Alkamides have been shown to be effective on CB2 (cannabinoid receptor type 2), which is thought to be one of the mechanisms behind their immunomodulatory characteristics [149,150,151]. Echinacea alkamides exhibit cannabinomimetic activities on two specific types of G protein-coupled receptors, namely, CB1 and CB2 cannabinoid receptors; this might be owing to structural similarities between them and anandamide, which is a natural cannabinoid receptor ligand.CB1 and CB2 receptors mediate anandamide's actions in the brain and peripherally. With several research projects, it has been discovered that the endocannabinoid signaling system's actions are not limited to the brain but are used all over the body [152,153]. The endocannabinoid system is a complicated signaling system that governs a wide range of physiological activities in central and peripheral nervous systems, including regulating and controlling allergic or inflammatory processes [154].

In contrast to the psychoactive effects of CB1 receptor agonists, drugs that act on CB2 receptors appear as promising drugs to fight inflammatory diseases. The CB2 receptor is located mainly in the periphery, especially in the blood cells and in the organs that produce blood cells [155]. One study showed that *Echinacea sp.* that is due to alkylamide derivatives binds even more strongly to the CB2 receptor than endogenous cannabinoids [152]. Recently, the results reconfirmed the agonist activity of alkylamide derivatives, which demonstrates selectivity for CB2 [156]. Other research found that TNF expression was revealed to be modulated by alkylamides found in Echinacea extracts. In human monocytes and macrophages, Echinacea extracts modulate mRNA through the CB2 receptor, as well as inhibition of stimulated LPS and TNF-α [157,158]. Based on these data, extracts derived from *Echinacea purpurea* (L.) Moench support the promise of anti-inflammatory and anti-pruritic benefits [156,159]. It is known that the endocannabinoid system regulates several parts of the immune functions and the skin barrier; therefore, targeting it may be a viable method of reducing the symptoms of atopic eczema [139].

Cannabinomimetic properties are also important for anxiolitic effect. Echinacea medicines were tested for anxiolytic efficacy among animals used in studies at reduced concentrations as compared to those that utilized unconventional applications. There is little information available about Echinacea and anxiety, but in the future, it will be possible to do research to prevent or even treat anxiety [159]. The former, when endogenous ligands activate it, takes an important role in the treatment of anxiety, while the latter is mostly involved immune system functions [160].Herbs high in alkamides cause paresthesia and have been used as toothache relief, antitussive and sialagogue by Native Americans in the past, as well as by physicians in the early 20th century [161].The alkamides have small structural variations, suggesting significant changes in CB receptor function; for example, the G-stimulating function was modified from reverse agonist to partial agonist when the isobutylamide fragment was replaced with a 2-methylbutylamide fragment [162]. The extracts of *Echinacea purpurea* (L.) Moench, on the other hand, were inactive in the antiacetylcholinesterase test [163]. White rats were used in a clofelin-induced depression test, where later the plant tincture improved the stimulating effect of L-DOPA and demonstrated antidepressant action [164].

#### 2.2.3. Antiviral Activity

In vitro, the aqueous extract of *Echinacea purpurea* (L.) Moench was effective against HSV-1 and HSV-2 (herpes simplex virus 1 and 2) strains that are acyclovir-resistant and acyclovir-susceptible [165] although the HSV-1 was suppressed by chicoric acid and hexane extract of the plant root [166]. Furthermore, chicoric acid inhibited the integrase of the human immunodeficiency virus type 1 (HIV-1) [167,168]. For 24 hours, mouse embryonic fibroblasts, after being incubated with alcoholic root extract and plant juice, were resistant to herpes, influenza A2 and vesicular stomatitis virus infection [169]. The avian strains (H7N7) and (H5N1), influenza viruses A (H1N1) and (H3N2) and the pandemic new swine-origin influenza (H1N1) in direct contact with the standard preparation of the plant demonstrated substantial inhibition. Moreover, hemagglutination (HA) experiments revealed that the preparation suppressed HA activity, implying that it might prevent a virus from entering treated cells [170]. Weight loss was detected in contaminated mice with influenza A H1N1 after administration of a plant polysaccharide extract, although lung virus titers were identical in treated and untreated animals. In treated animals, lower levels of keratinocyte chemoattract (KC), interleukin 10 (IL-10), and systemic interferon-gamma (IFN) were found, suggesting that EP might influence the clinical symptoms of influenza via modulating cytokines [171]. According to the findings of the recent research studies, distinct plant components appear to have positive effects on influenza patients via various mechanisms [172]. Therefore, to be identified as a biological agent, more study is needed, particularly in vivo studies and research on the toxic effects of these compounds.

There are already clinical trials of SARS-CoV supporting the use of EP against the coronavirus. Preparations of *Echinacea purpurea* (L.) Moench may be efficient as preventative therapy for all CoVs because of their structural similarities, but further studies are needed [173,174,175,176,177,178,179,180,181,182,183].

#### 2.2.4. Antimicrobial Effect

According to recent research, several standardized formulations exhibit potent and selective antiviral and antibacterial properties. Numerous investigations on the effects of *Echinacea purpurea* (L.) Moench formulations have lately been done in an endeavor to substantiate a few of the more common uses of EP. The Hudson JB study's findings revealed that EP and other Echinacea extracts have selective antibacterial activity, that different creatures had their sensitivity patterns varied significantly and that there were no correlations between the chemical components of the extracts, as measured by known marker compounds, and their antimicrobial activities. In addition, various preparations of EP have noticeably shown various bacterial impacts, demonstrating that EP has various antibacterial actions against each pathogen. Purple coneflower can generally overturn proinflammatory cytokine stimulation, regardless of the bacteria or virus that is causing the infection [184,185]. Several groups investigated the effects of *Echinacea purpurea* (L.) Moench on the activation of LPS (inflammatory mediators by bacterial lipopolysaccharide) (typically produced from *Escherichia coli*) in various cultures of human cells and animals, in addition to research using live bacteria. Although such models may not always mimic living bacteria, they can be helpful in testing for anti-inflammatory drugs. These findings suggest that EP is a general anti-inflammatory drug capable of alleviating several of the symptoms of respiratory infections [186,187]. The extract derived from the aerial elements of *Echinacea purpurea* (L.) Moench has higher antibacterial and antioxidant properties than the extract acquired by ultrasonic extraction [4]. Antibiotics and vaccinations are currently used to treat bacterial infections as prophylactic measures. In many circumstances, the management of respiratory infections is a critical issue because of several microorganisms in the disease. Using phytopreparations throughout this case might be a viable option [188,189].

To gain a greater knowledge of the synergistic interactions of Echinacea extracts with antibiotics, further studies are needed to uncover the mechanism behind their antimicrobial action and then to identify numerous routes that may be targeted. 

#### 2.2.5. Antioxidant Activity

Several reports have demonstrated the antioxidant properties of *Echinacea purpurea (L.) Moench*. Therefore, during infections, EP may give extra protection by maintaining the normal redox status [190,191].

*Echinacea purpurea* (L.) Moench 70% aqueous ethanolic extracts are a possible source of bioactive natural and non-toxic chemicals that serve as antioxidants. The extract prepared by standard extraction from EP aerial sections had a higher concentration of bioactive components [4]. The antioxidant property of *Echinacea purpurea* (L.) Moench, using the aerial part, is made up of bioactive polyphenolic components. Polyphenols are antioxidants obtained from plants, which have photoprotective, anticarcinogenic and anti-inflammatory properties [192,193,194]. All of these therapeutic properties of the extract of *EP*, using the aerial parts, are beneficial in the cosmeceutical efficiency to prevent the aging of the skin. Antioxidants are compounds that can stop the oxidation reaction triggered by the use of free radicals. They can minimize skin injury by neutralizing the damaging effects of free radicals and reactive oxygen species [195,196].

Significant antioxidant capacity measurements are broadly classified into two types: hydrogen atom transfer (single-electron transfer reaction-based assays and H reaction-based assays) [197]. The phenolic content and phenolic profiles of the extracts, as well as their antioxidant activity, may all be analyzed. The following methods can be used: scavenging of DPPH radicals, ABTS, FRAP, the Folin–Ciocalteu colorimetric method, voltametry—a sensitive electrochemical method, CUPRAC [198,199,200,201,202,203,204,205,206,207,208,209]. Antioxidant activity can be measured using any of the methods.

#### 2.2.6. Antiosteoporotic Activity

Current studies highlight scientific information about the natural chemical compounds obtained from herbal remedies that have anti-osteoporosis or anti-osteonecrosis qualities. The emphasis is on international literature published during the previous 10 years [210]. *Echinacea* sp. contains echinacoside, which is a phenylpropanoid glycoside that was isolatedto produce substantial increases in alkaline phosphatase activity, MC3T3-E1 cell proliferation, osteocalcin levels, collagen 1 secretion and mineralization when isolated from *Cistanche tubulosa*. The scientists found that osteoprotegerin (OPG) and receptor activator of nuclear factor kappa-B ligand (RANKL) may have a role in echinacoside's anti-osteoporotic activity. An in vivo follow-up study demonstrated the effects of echinacoside in counteracting the damage caused by ovariectomy, leading to improved bone mineral density and bone biomechanical properties [172]. Most osteoporotic patients are postmenopausal women and estrogen deficiency is the predominant cause of rapid hormone-related rapid bone loss after menopause. Natural alternatives are being investigated in this area to avoid bone loss and the danger of fracture caused by osteoporosis, as well as less desirable side effects [211]. Anti-inflammatory and antioxidant characteristics are linked to several osteoclast suppressants. According to Aarland et al. [156], *Echinacea purpurea* (L.) Moench has been already explored for its antioxidant and anti-inflammatory properties. Therefore, it has also been thought to help with inflammation-related osteoclastogenesis. The accidental roots of EP were fractionated utilizing various chromatographic methods, yielding three novel chemicals and eight recognized compounds. Antioxidant and anti-inflammatory action were initially assessed to identify a novel chemical capable of suppressing osteoclasts. Among the new compounds, echinalkamide (compound 1, isobutylamide of undeca-2Z-4E-diene-8,10-diinoic acid) shows the best antioxidant and anti-inflammatory benefits. The possibility of echinalkamide as an osteoporosis therapy, as well as the mechanism behind it, has been evaluated [212]. According to the findings, echinacoside improves bone regeneration by boosting OPG ratios and promoting cell signaling pathways. The research indicates that echinacoside and echinalkamide enhance cell proliferation, differentiation and mineralization by modulating the stimulus of OPG and signaling pathways. As a result, *Echinacea purpurea* (L.) Moench is a potential herbal medication for osteoporosis prevention and treatment.

A pharmaceutical preparation in the form of capsules containing *Echinacea sp.* in conjunction with *Gentiana sp*. has become available on the pharmaceutical market and can be prescribed for the prevention of osteoporosis.

Given the potential in pharmaceutical applications, EP has attracted the interest of many pharmaceutical scientists, but further studies are needed to interpret the molecular mechanisms and clinical trials in the treatment of osteoporosis.

## 3. Conclusions

Studies on the species *Echinacea purpurea* (L) Moench have discovered a large range of bioactive compounds, indicating that it is a rich source of phytochemicals that might be used to cure a number of diseases. Antioxidants, immunomodulators, anti-inflammatory, antibacterial, antiviral and antiosteoporotic are just a few of the positive biological effects that indicate how important this species is in the ecosystem. There are differences in the extracts' phytochemical, biological and pharmacological properties that come from different sources. The findings of the investigations show that standardization measures are needed to ensure not only the safety and identification of botanical products but also their pharmacological effectiveness. EP is a source of bioactive chemicals, but only a tiny portion of its characteristics have been studied, and some of them might be valuable therapeutic tools. For future perspectives, it is important to expand the studies on the dermatological effects. There are real chances for the development of EP-based products, effective in various common dermatological conditions, that are due to bioactive compounds. At the same time, further research into the in-depth understanding of the cannabinomimetic properties has major importance for the development of new pharmaceuticals.

## Figures and Tables

**Table 1:** Correlation between the storage condition and bioactive compounds concentration.

Storage Condition	Storage Temperature	Alchylamides Concentration	Chicoric Acid Concentration	Ref.
60 days in the dark	5 °C	unchanged	70% decrease	[71,72]
60 days in the light	20 °C	65% decrease	unchanged	[71,72]

**Table 2:** Biological and pharmacological effects of the bioactive compounds of *Echinacea purpurea* (L.) Moench.

Bioactive Compounds	Biological and Pharmacological Effects	References
Alkylamides	Anti-inflammatory	[74,75,76,77,78,79,80,81]
	Immunomodulatory	
	Modulation of macrophages	
	Reduction of NO and tumor necrosis factor -α	
	Mediators of antiviral immunity	
	Cannabinoid receptor type 2	
Polysaccharides	Antitumoral	[82,83,84,85,86,87,88,89,90,91,92,93,94,95,96,97,98]
	Antioxidant	
	Antimicrobial	
	Antifungal	
	Antiviral	
	Immunomodulatory	
	Hypoglycemic	
	Hepatoprotective	
	Gastrointestinal-protective	
	Antidiabetic	
Glycoproteins	Immunomodulatory	[99,100,101,102,103]
Flavonoids	Antioxidant activity	[104,105,106,107,108,109]
	Anti-inflammatory	
	Anti-ulcer activity	
	Antiallergic	
	Antiviral	
Caffeic acid derivatives	Antioxidant activity	[110,111,112,113,114,115,116,117,118,119,120,121,122,123,124,125,126]
	Antiosteoporotic activity	
	Anti-inflammatory	
	Antimicrobial	
	Anti-tumoral	
	Neuroprotective action	

## Data Availability

Not applicable.
